# The Impact of SsPI-1 Deletion on *Streptococcus suis* Virulence

**DOI:** 10.3390/pathogens8040287

**Published:** 2019-12-06

**Authors:** Yan Zhao, Gang Li, Xin-Yue Yao, Shu-Guang Lu, Jing Wang, Xiao-Dong Shen, Ming Li

**Affiliations:** 1 Department of Microbiology, College of Basic Medical Sciences, Army Medical University (Third Military Medical University), Key Laboratory of Microbial Engineering under the Educational Committee in Chongqing, Chongqing 400038, China; hnyanyanxp@aliyun.com (Y.Z.); liganglg1990@163.com (G.L.); shulang88@126.com (S.-G.L.); wj.jz.cq@gmail.com (J.W.); 2 Jinling Hospital Research Institute of Clinical Laboratory Medicine, Nanjing University, School of Medicine, Nanjing 210002, China; xinghai0903@aliyun.com; 3 Department of Biochemistry and Molecular Biology, College of Basic Medical Sciences, Army Medical University (Third Military Medical University), Chongqing 400038, China

**Keywords:** *Streptococcus suis*, pathogenicity island, deletion, virulence

## Abstract

(1) Background: *Streptococcus suis* is an important zoonotic pathogen that infects pigs and can occasionally cause life-threatening systemic infections in humans. Two large-scale outbreaks of streptococcal toxic shock-like syndrome in China suggest that the pathogenicity of *S. suis* has been changing in recent years. Genetic analysis revealed the presence of a chromosomal pathogenicity island (PAI) designated SsPI-1 in Chinese epidemic *S. suis* strains. The purpose of this study is to define the role of SsPI-1 in the virulence of *S. suis*. (2) Methods: A SsPI-1 deletion mutant was compared to the wild-type strain regarding the ability to attach to epithelial cells, to cause host disease and mortality, and to stimulate host immune response in experimental infection of piglets. (3) Results: Deletion of SsPI-1 significantly reduces adherence of *S. suis* to epithelial cells and abolishes the lethality of the wild-type strain in piglets. The SsPI-1 mutant causes no significant pathological lesions and exhibits an impaired ability to induce proinflammatory cytokine production. (4) Conclusions: Deletion of the SsPI-1 PAI attenuates the virulence of this pathogen. We conclude that SsPI-1 is a critical contributor to the evolution of virulence in epidemic *S. suis*.

## 1. Introduction

*Streptococcus suis* is a common swine pathogen and a major zoonotic threat to people working in close contact with infected pigs or pork products [1]. Unlike the various serotypes infecting pigs, *S. suis* serotype 2 remains the most common cause of human *S. suis* cases [2]. Historically, most human infections with *S. suis* are sporadic cases of meningitis and septicaemia [3,4,5]. However, two large human outbreaks associated with an unusual streptococcal toxic shock-like syndrome (STSLS) have emerged in China [6,7], allowing people to re-recognize this newly evolved pathogen. Although considerable progress has been made in elucidating the pathogenesis of *S. suis* infections during the last decade, there are significant knowledge gaps that need to be addressed to deepen our understanding of the biology, ecology, and pathogenesis of this pathogen, toward the development of new strategies for prevention and control of *S. suis* infections.

It was reported that *S. suis* is the species with the greatest level of positive selection pressure within the genus *Streptococcus*, displays the highest rates of gene gain and loss, and presents greatest amount of genomic structure variation [8]. Correspondingly, *S. suis* isolates can have three different pathogenic phenotypes: high-pathogenic, low-pathogenic, and non-pathogenic (avirulent) [9]. To determine whether the high pathogenicity of the emerging Chinese epidemic isolates is associated with genotypic variation, whole genome sequencing and comparative genomics analyses were carried out, revealing that the epidemic strains characteristically harbour a unique pathogenicity island (PAI) designated 89K (later renamed SsPI-1) that is responsible for the two outbreaks [10,11]. PAIs are typically recognized as distinct regions of chromosomally integrated DNA acquired by horizontal gene transfer, which contribute significantly to bacterial evolution and to the emergence of new pathogenic variants [12,13]. Further genetic analysis confirmed that SsPI-1 can laterally transfer to recipient strains and, thus, potentially make them more virulent [14,15]. These factors are important in the emergence, dissemination, and prevalence of epidemic *S. suis* strains.

In a previous work, we demonstrated that an SsPI-1-borne toxin–antitoxin system (designated SezAT) acts as a stabilization factor assuring inheritance of SsPI-1 during cell division by killing of any descendants that are free of this element, which may drive the persistence of prevalent *S. suis* [16]. When devoid of a functional SezAT system, we successfully cured the entire SsPI-1 PAI from the host strain using the Cre-*loxP* site-specific recombination system. In this study, to evaluate the role of SsPI-1 in the pathogenesis of *S. suis* infection, we assessed the impact of SsPI-1 deletion on the virulence of *S. suis* in vitro and in vivo. Cellular and animal experimental results showed that elimination of SsPI-1 significantly impairs the pathogenicity of this pathogen, suggesting that SsPI-1 plays a vital role in the virulence of *S. suis*.

## 2. Results

### 2.1. Elimination of SsPI-1 Does Not Affect the General Biological Characteristics of *S. suis*

**Figure 1:**
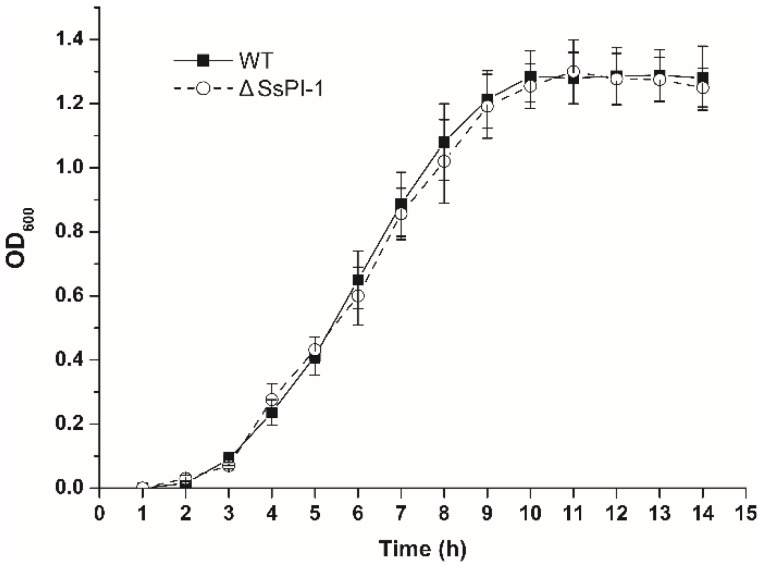
Growth curves of wild-type (WT) and the ΔSsPI-1 mutant. *Streptococcus suis* strains were cultured in liquid Todd–Hewitt broth supplemented with 2% yeast extract (THY) medium at 37 °C. Absorbance at 600 nm was measured each hour. Data were analysed using the Student’s *t*-test and presented as means ± standard deviations (SD).

Prior to evaluating the effect of SsPI-1 deletion on the virulence of *S. suis*, we first compared the growth characteristics of the wild-type (WT) and ΔSsPI-1 mutant strains. As shown in Figure 1, both strains exhibited similar growth kinetics. Haemolytic phenotype and cell morphology were also examined; however, no detectable differences were observed between the two strains (data not shown).

### 2.2. Deletion of SsPI-1 Significantly Impairs the Adhesion Ability of *S. suis*

**Figure 2:**
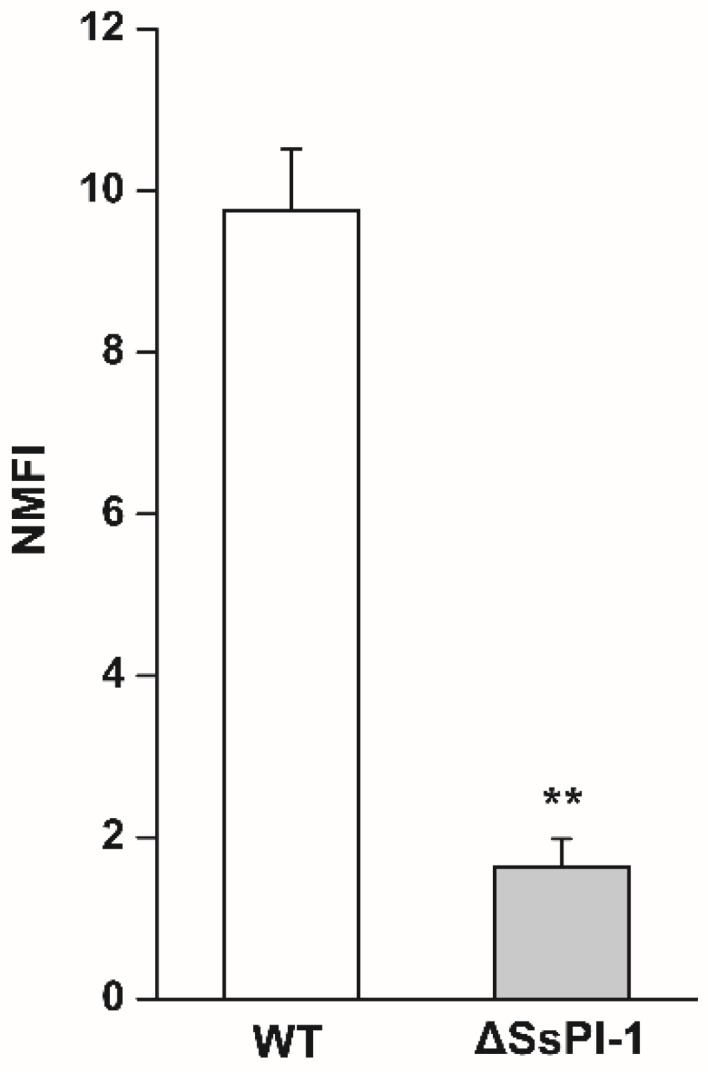
Adherence of the ΔSsPI-1 mutant to Hep-2 cells compared with the wild-type (WT) strain. Data were analysed using the Student’s *t*-test and the normalized mean fluorescence intensities (NMFI) of Hep-2 cells after incubation with carboxyfluorescein diacetate succinimidyl ester (CFDA-SE)-labelled *S. suis* are shown as means + SD (** indicates *p* < 0.01).

After staining with carboxyfluorescein diacetate succinimidyl ester (CFDA-SE), a significant increase in fluorescence intensity was observed for labelled bacteria compared to unlabelled bacteria (Supplementary Figure S1), suggesting that *S. suis* cells were effectively labelled with CFDA-SE. Following co-incubation of Hep-2 epithelial cells with fluorescently labelled bacteria and flow cytometry analysis, the normalized mean fluorescence intensities (NMFI) were calculated, and the results showed that ΔSsPI-1 had significantly decreased adherence to Hep-2 cells compared with the WT strain (Figure 2).

### 2.3. SsPI-1 Is Essential for the Full Virulence of *S. suis*

**Figure 3:**
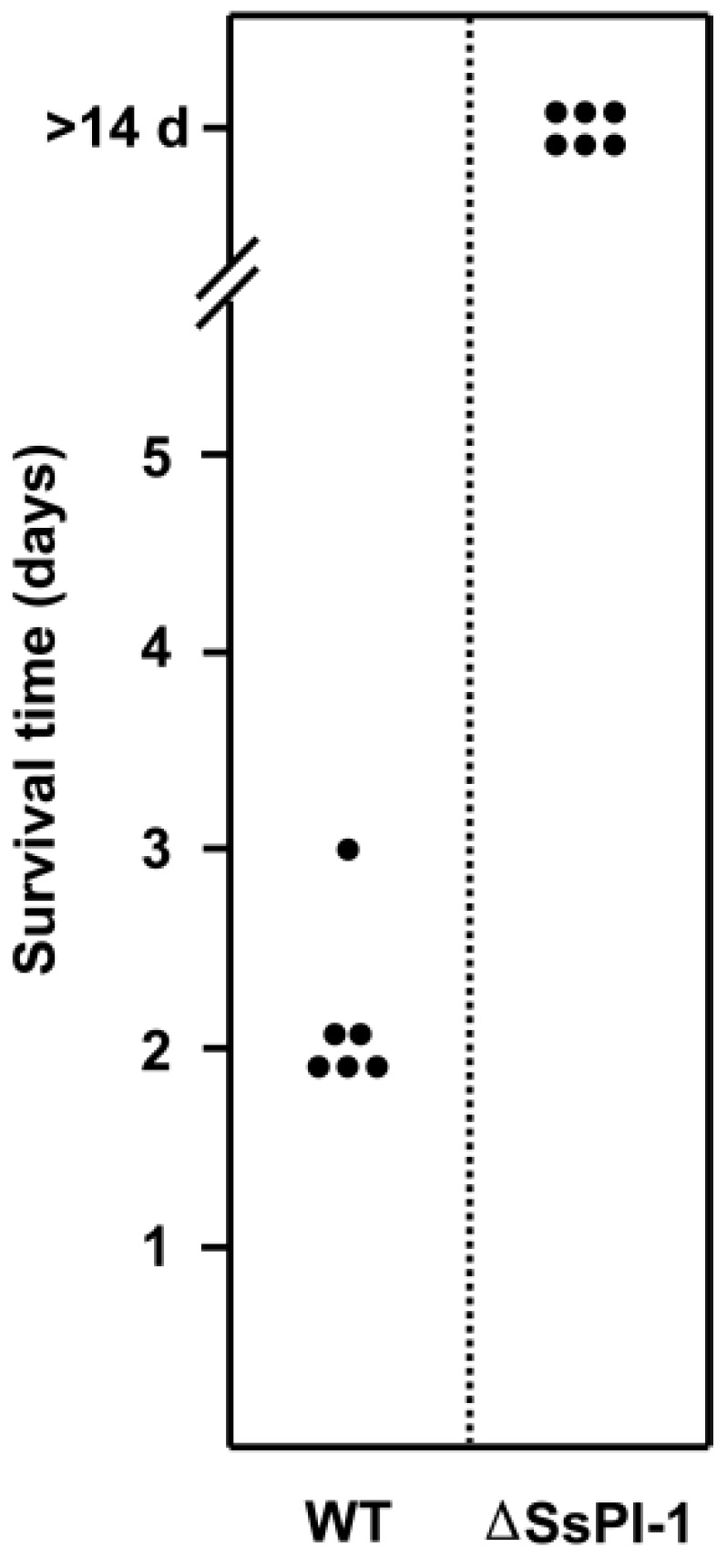
SsPI-1 deletion abolishes the lethality of the wild-type (WT) strain of *S. suis*. Groups of six piglets were challenged intravenously with a dose of 10^8^ colony forming units (CFU) of WT or the ΔSsPI-1 mutant. Survival time of individual piglets is indicated.

In the in vivo experimental infection studies, all piglets challenged with WT and the ΔSsPI-1 mutant had positive blood cultures, revealing that *S. suis* cells have invaded the bloodstream. The six WT-infected piglets developed specific symptoms of *S. suis* infection, including high fever, poor appetite, shivering, swollen joints, limping, and respiratory distress within 24 h. Five piglets died two days post-infection, and the remaining one died on day 3 (Figure 3). In stark contrast, all piglets in the ΔSsPI-1 mutant group survived the challenge without developing obvious symptoms during the entire monitoring period. 

**Figure 4:**
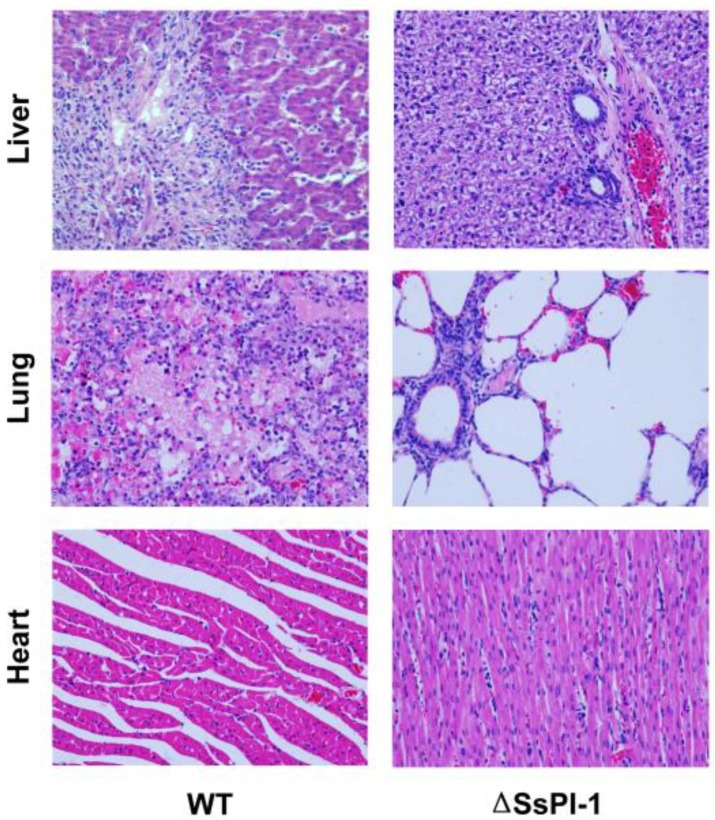
Pathological examination of vital organs of infected piglets on day 2 post-challenge. Morphological changes were viewed by light microscopy (hematoxylin-eosin staining, ×400 magnification).

In the next set of animal experiments, samples were taken from WT-infected piglets developing typical symptoms on day 2 post-infection, histological examination of the liver tissue revealed swelling of the hepatocytes and fibroplasia in the portal area. Focal and piecemeal necroses with massive infiltration of eosinophils, macrophages, and lymphocytes were observed throughout the liver (Figure 4). Typical histopathological changes in lung tissues included extensive consolidation, enlarged alveolar septa with blood vessel dilatation and congestion. Alveolar spaces were diffusely filled with oedema fluid and fibrin exudates admixed with large numbers of macrophages and eosinophils. Moreover, pathological examination of the heart revealed the presence of diffuse myocardial interstitial oedema. By contrast, no significant pathological changes were observed in these tissues collected parallelly from a representative piglet inoculated with the ΔSsPI-1 mutant, except for slight infiltration of lymphocytes in the portal area of the liver (Figure 4). 

### 2.4. Elimination of SsPI-1 Markedly Decreased Proinflammatory Cytokine Production In Vivo 

**Figure 5:**
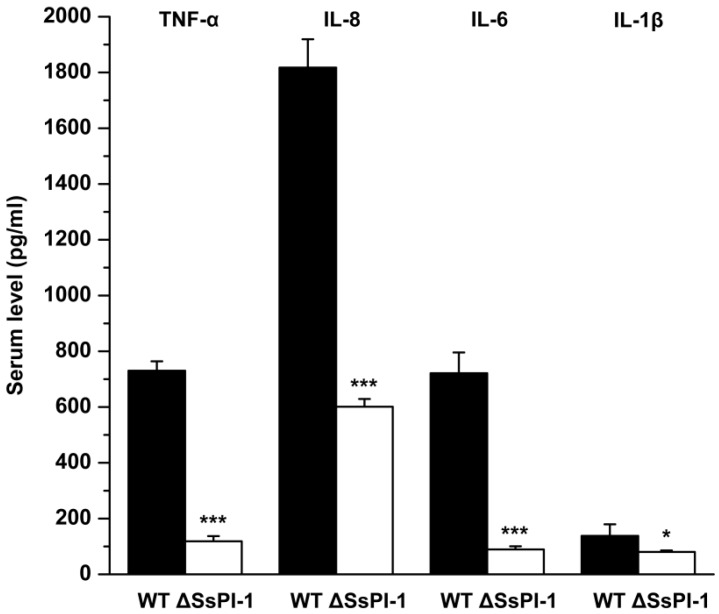
Serum levels of cytokines in piglets infected with the wild-type (WT) and ΔSsPI-1 mutant strains. EDTA-anticoagulated blood samples were collected from the precaval vein of piglets that developed specific symptoms of *S. suis* infection (on day 2 post-challenge) and centrifuged in preparation for cytokine concentration measurements using enzyme-linked immunosorbent assay (ELISA) kits (R&D Systems). Statistical significance is calculated using the Student’s *t*-test (*: *p* < 0.05; ***: *p* < 0.001). Data are expressed as means + SD.

As shown in Figure 5, production of tumour necrosis factor-α (TNF-α), interleukin-6 (IL-6), interleukin-8 (IL-8), and interleukin-1β (IL-1β) were significantly decreased in piglets infected with the ΔSsPI-1 mutant compared with those infected with the WT strain, suggesting that deletion of SsPI-1 impairs its capacity to induce proinflammatory cytokine responses within the host or indirectly as a consequence of reduced bacteraemia and, thus, antigenic charge.

## 3. Discussion

Previous studies have shown that the SsPI-1 PAI is only present in the highly virulent Chinese *S. suis* isolates [10,11], and might have been acquired by horizontal gene transfer [15], which may confer pathogenicity to the host strain. In the present work, we demonstrated that deletion of SsPI-1 significantly reduced bacterial adherence to epithelial cells and abolished the lethality of *S. suis* in a piglet infection model. As well as causing fewer pathological lesions, the ΔSsPI-1 mutant also has a decreased ability to elicit a host response to induce cytokine production. Taken together, these results provide supporting evidence of a vital role for SsPI-1 in the pathogenesis of *S. suis*. 

The loss of virulence in the SsPI-1-free variant can probably be attributed to the concomitant absence of several known important virulence-associated factors encoded within the island that are closely related to the pathogenesis of *S. suis* infection. For example, the SalK/SalR two-component system (TCS) located in the centre of the SsPI-1 island is a crucial regulator both at the transcriptional and protein level [17], and was demonstrated to be essential for full virulence of highly pathogenic *S. suis* strains [18]. An NisK/NisR TCS encoded at the 3’ terminal region of SsPI-1 was also reported to play an important role in the process of colonization and invasion, thus contributing to bacterial pathogenicity [19]. The type IV secretion system (T4SS) encoded at the 5’ end of SsPI-1 can not only mediate the conjugal transfer of SsPI-1 [15], but also deliver effectors directly into the host cell, thus causing substantial tissue damage [20]. Furthermore, comparative genomics analyses revealed the complete absence of SsPI-1 in a native avirulent strain 05HAS68, but this strain contains instead, a novel ~113 kb metabolic island in the corresponding position of the genome [21], underscoring again the critical role of SsPI-1 in the pathogenesis of *S. suis*.

At present, how the epidemic *S. suis* strains cause STSLS still remains unclear. A retrospective clinical study revealed that serum levels of proinflammatory cytokines were significantly more elevated in patients with STSLS than in those with meningitis only [22]. It later became established that the onset of a cytokine storm was essential for STSLS development and the associated high mortality [23,24,25]. Since the SsPI-1 PAI is specifically present in the genome of the epidemic *S. suis* strains but not in other clinical isolates [11], it is reasonable to propose that acquisition of SsPI-1 enables the strain to have the ability to cause a higher level of inflammatory cytokine production and subsequent STSLS development. Support for this assumption comes from the observation that several gene products within SsPI-1 were found to be closely associated with the occurrence of STSLS. The aforementioned T4SS encoded within was recently proved to be able to secrete a potential virulence effector named SspA-1 [26], which can trigger an intense inflammatory response both in vivo and in vitro. In another study, a new proinflammatory effector designated PrsA secreted by T4SS under oxidative stress was also identified to increase expression of proinflammatory cytokines in murine macrophage cells [27]. Moreover, a transcriptional regulator TstS harboured in SsPI-1 was demonstrated to stimulate cytokine production and bacteraemia to promote STSLS [28]. Strikingly, a haemolysis-related gene located in the SsPI-1 PAI was previously reported to increase the expression of suilysin (SLY) [29], which was the main *S. suis* protein that stimulates inflammation independently of its haemolytic ability [30]. Very recently, SLY was confirmed to be fully responsible for the high level of NLRP3 inflammasome activation in response to *S. suis* infection [31], which was essential for induction of the cytokine storm and multiple organ dysfunction—the hallmarks of STSLS. Nevertheless, reduced inflammation could also be related to reduced invasive capacity of the mutant strain and, thus, reduced overall levels of bacteria in blood and organs.

Current control of *S. suis* infection is still hampered by the lack of reliable diagnostics as well as safe and effective vaccines, due to the complexity of *S. suis* epidemiology [32]. Serotype-specific bacterins have no prospects of developing a protective vaccine against *S. suis*, and many subunit vaccine candidates have been evaluated, but only few give hope to the discovery of a universal vaccine. Vaccines under development are expected to reduce mortality and clinical manifestations of *S. suis* systemic disease. In a previous experimental study, a native avirulent *S. suis* strain of serotype 2 isolated from the tonsil of a healthy pig was shown to provide complete protection against a lethal homologous *S. suis* serotype 2 challenge in piglets [21], which has implications for the development of an efficacious vaccine to fight this important pathogen. Similarly, a good protection was also observed in mice and swine when using another live avirulent *S. suis* type 2 strain (#1330), which was first isolated from the lung of a pig with pneumonia [32]. In this study, the SsPI-1 deletion mutant was determined to be also avirulent in a piglet infection model, and its ability to induce a protective response needs to be explored further. Work currently underway in our laboratory seeks to define its potential as a vaccine candidate against *S. suis* disease.

## 4. Materials and Methods

### 4.1. Bacterial Strains and Growth Conditions

*S. suis* strain 05ZYH33 was isolated from a dead patient with STSLS during the 2005 outbreak in China and kept in our laboratory. ΔSsPI-1 was a derivative of 05ZYH33 with the entire SsPI-1 sequence replaced by a single 34 bp *loxP* site [16]. Whole-genome sequencing and PCR analysis confirmed that no mutations have accumulated outside the *loxP* site during the site-specific recombination. *S. suis* strains were cultured at 37 °C in Todd–Hewitt broth (THB) supplemented with 2% yeast extract (THY) or seeded on 5% (vol/vol) sheep blood agar plates.

### 4.2. Cell Adhesion Analysis

Human laryngeal epithelial cell line Hep-2 (CCTCC GDC004) was used to test adhesion ability of *S. suis* strains as described previously [33]. Briefly, mid-exponential-phase bacteria were harvested, washed, and resuspended in phosphate buffered saline (PBS) containing 10 μM CFDA-SE for 20 min. Labelled bacteria and Hep-2 cells were co-incubated (at 100:1 ratio) for 2 h at 37 °C. Cells were washed and then fixed with 4% paraformaldehyde before flow cytometry analysis. The normalized mean fluorescent intensity (NMFI) values of the cells after incubation with the bacteria were calculated. 

### 4.3. Experimental Infections of Piglets

To evaluate the effect of SsPI-1 deletion on the pathopoiesis of *S. suis* in vivo, virulence studies were conducted using an experimental infection model in piglets as we previously described [18]. Groups of six three-week-old piglets were challenged intravenously with 10^8^ colony forming units (CFU) of the WT or ΔSsPI-1 mutant. Blood cultures were conducted to qualitatively detect bacterial invasion of the bloodstream. When piglets developed typical signs of *S. suis* infection or became mortally ill, they were anesthetized with 4% isoflurane and euthanized with CO_2_ inhalation. Samples of the lung, liver, heart, spleen, and kidney were taken and fixed in 4% paraformaldehyde for histopathological examination. All animal experiments were approved by the Laboratory Animal Welfare and Ethics Committee of the Third Military Medical University and complied with institutional animal welfare and ethical guidelines.

### 4.4. Enzyme-Linked Immunosorbent Assay (ELISA)

EDTA-anticoagulated blood samples were collected from the precaval vein of infected piglets developing specific symptoms of *S. suis* infection and centrifuged at 10,000 × g for 10 min to obtain plasma. Levels of TNF-α, IL-6, IL-8, and IL-1β in plasma were determined using the enzyme-linked immunosorbent assay (ELISA) Kits (R&D Systems) according to the manufacturer’s instructions. 

### 4.5. Statistical Analysis

All assays were biologically repeated more than three times independently. Data were analysed using the Student’s *t*-test. Differences were considered statistically significant at *p* < 0.05.

## 5. Conclusions

In conclusion, the results presented here demonstrated the crucial role of SsPI-1 in the pathogenesis of epidemic *S. suis* strains, thus contributing to a better understanding of the evolution of virulence in this important zoonotic bacterium.

## Supplementary Materials

The following are available online at https://www.mdpi.com/2076-0817/8/4/287/s1, Figure S1: Labelling of *S. suis* cells with CFDA-SE.
