# Supplementary material for: The Impact of SsPI-1 Deletion on Streptococcus suis Virulence
Source: Pathogens. 2019 Dec 6;8(4):287. doi: 10.3390/pathogens8040287 (PMC6963714; doi:10.3390/pathogens8040287)
Supplement: Supplementary file 1 [file pathogens-08-00287-s001.pdf]

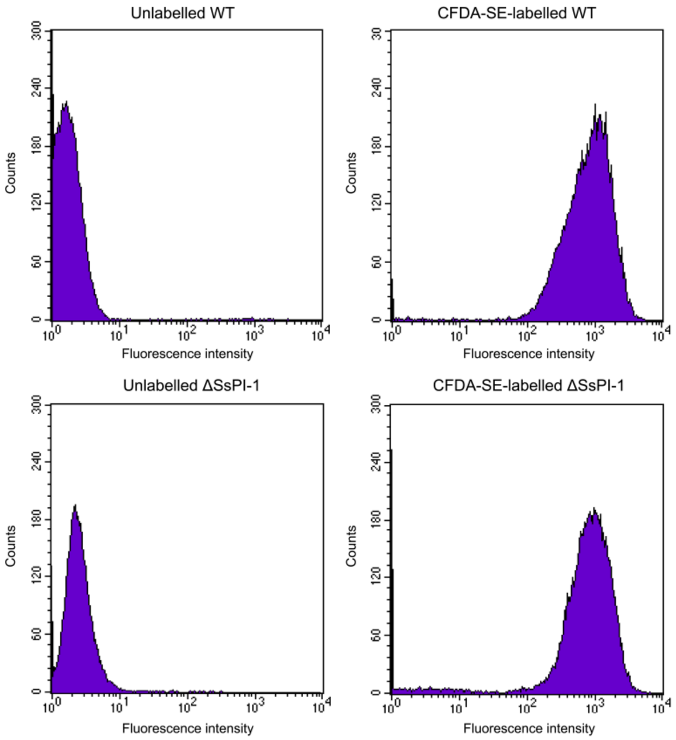

Supplementary Figure S1. Labelling of *S. suis* cells with CFDA-SE. Mid-logarithmic-phase bacteria were washed and labelled with CFDA-SE for 20 min at 37° C, then subjected to flow cytometer analysis. The labelling of the bacteria is seen as a shift in fluorescence intensity of the bacteria.
